# A situational analysis of child and adolescent mental health services and systems in the Western Cape Province of South Africa

**DOI:** 10.1186/s13034-022-00440-7

**Published:** 2022-01-25

**Authors:** Stella Mokitimi, Marguerite Schneider, Petrus J. de Vries

**Affiliations:** 1 Division of Child and Adolescent Psychiatry, University of Cape Town, 46 Sawkins Road, Rondebosch, 7700 South Africa; 2 Red Cross War Memorial Children’s Hospital, Klipfontein Road, Rondebosch, 7700 South Africa; 3 Alan J Flisher Centre for Public Mental Health, Department of Psychiatry and Mental Health, University of Cape Town, 46 Sawkins Road, Rondebosch, South Africa

**Keywords:** Situational analysis, Child, Adolescent, Mental health, Services, Western Cape, South Africa, Low- and middle-income countries, LMIC

## Abstract

**Background:**

Even though child and adolescent mental health is a global health priority, services are very limited, particularly in low- and middle-income countries (LMIC), and therefore need comprehensive strengthening. This requires knowledge of the hardware elements of the system (human resources, financing, medicines, technology, organisational structure, service infrastructure, and information systems). This study sought to examine these elements of child and adolescent mental health (CAMH) services and systems in the Western Cape Province of South Africa.

**Methods:**

The World Health Organization Assessment Instrument of Mental Health Systems (WHO-AIMS) version 2.2 of 2005 was adapted to identify key variables of interest in CAMH. Data were collected for the calendar year 2016 and focused on the public health sector. We outlined findings based on best available data across the six domains of the WHO-AIMS.

**Results:**

In domain 1, we found no provincial CAMH policy or implementation plans to support the national CAMH policy and were unable to identify a CAMH-specific budget. In domain 2, there was no dedicated provincial leadership structure for CAMH, and no dedicated or ‘child- and adolescent-friendly’ mental health services at primary or secondary care levels. At tertiary level, there were only three specialist CAMH teams. The majority of CAMH resources were based in the City of Cape Town, with limited resources in the rural districts. Essential medicines were available in all facilities, and the majority of children and adolescents had access to free services. In domain 3, data were limited about the extent of training offered to primary healthcare staff, and little or no psychosocial interventions were available in primary care. Domain 4 identified a small and variable CAMH workforce across all levels of care. In domain 5, few public health campaigns focused on CAMH, and little evidence of formal intersectoral collaboration on CAMH was identified. Domain 6 identified significant limitations in health information systems for CAMH, including lack of child- and adolescent-specific and disaggregated data to establish baselines for policy development, monitoring, evaluation and CAMH research.

**Conclusions:**

This study identified significant structural weaknesses in CAMH and presents a clear call for action to strengthen services and systems in the province and in South Africa. it would be important to expand research also to include provider and user perspectives for service strengthening.

## Background

Child and adolescent mental health is a global health priority, yet it is generally known that services are limited, particularly in low- and middle-income countries (LMIC) [1, 2]. Health system strengthening requires an understanding of multiple landscapes: the policy and resource landscape (representing mostly hardware elements of health systems), and the landscape as perceived by stakeholders (which would describe both hardware and software elements of the healthcare system) [3, 4]. Hardware typically refers to human resources, financing, medicines, technology, organisational structure, service infrastructure, and information systems, while the software typically refers to ideas and interests, relationships and power, values and norms, and the interactions between all factors and actors [3, 4].

As stated by the World Health Organization (WHO) [4] and summarised by Gilson [3], all the components of a health system should be balanced in order for the system to be responsive to the needs of the community it serves. A good enough health system should have sufficient resources that are equitably distributed, and human resources that have sufficient competencies to respond to the needs of the population [4].

The geographical focus of our work is South Africa, an upper-middle-income country that has some of the greatest economic and health disparities in the world [5, 6]. A situational analysis conducted by Kleintjes and colleagues in 2005 [2] evaluated key aspects of child and adolescent mental health (CAMH) services and systems in South Africa, Uganda, Zambia and Ghana. In comparison to the other three sub-Saharan African countries, South Africa had relatively more CAMH resources [2]. However, only 1.4% of all mental health outpatient services in the country, 3.8% of mental health beds in general hospitals, 1% of specialist mental health hospital beds, and 1% of day patient facilities in the country were dedicated to CAMH. There were no specialist CAMH hospitals. The number of psychiatrists (general and child & adolescent psychiatrists combined) was estimated at 0.28 per 100,000. No data were available on the number of mental health professionals in schools [7]. Qualitative data from semi-structured interviews with key stakeholders in the Kleintjes study [2] proposed three main themes as the reasons for the very low resources for CAMH: first, the impact of stigma associated with mental health disorders; second, the low priority of all aspects of mental health in LMIC; and third, the lack of attention to the link between poverty and poor mental health [2]. While these proposed reasons are understandable when comparing mental health with physical health services, Kleintjes and colleagues did not attempt an explanation for the under-representation of child and adolescent versus adult mental health services.

Since the situational analysis by Kleintjes and colleagues [2], Babatunde and colleagues studied one specific rural health district (Amajuba district) in one South African province (KwaZulu-Natal) [8]. Apart from this important focused study, no other study has conducted a fine-grained situational analysis of CAMH services and systems in South Africa or any other LMIC in the last decade.

### The Western Cape Province of South Africa

In order to perform a more detailed situational analysis of CAMH, we selected the Western Cape as the focus for our work. The Western Cape was selected for two reasons. Firstly, it is one of the provinces with better resources in terms of health services [9, 10] and therefore more accessible for research scrutiny in terms of data sources, documents, and dedicated staff for mental health. Secondly, it is the base of our own clinical activities in the Division of Child and Adolescent Psychiatry (DCAP) at the University of Cape Town. It therefore represents the immediate and direct health system and services in which we are actors, and gives us a strong knowledge and experience-base from which to examine and interpret available data.

In 2016, the year selected for data collection, South Africa had an overall estimated population of 55.9 million [11]. Healthcare in South Africa is provided in two parallel systems. Approximately 20% of the population have private health insurance and therefore have access to private healthcare. The remaining ~ 80% of the population do not have private health insurance, and are dependent on government/state-funded healthcare. These services are provided free of charge or at a significantly lower rate than in the private sector, based on the income of service users. In South Africa (as in the majority of low/middle-income countries), government healthcare services are modelled on the World Health Organization (WHO) three-tier system (primary, secondary and tertiary). Primary healthcare services include three care settings—home and community-based care (HCBC), primary care (provided at primary healthcare clinics) and intermediate care (provided at intermediary care centres). Hospital-based care is provided by district hospitals (based in health districts), regional hospitals (covering a larger region of health districts), and at tertiary and central hospitals (based in large cities and linked to university teaching centres). CAMHS are provided across all three these levels of care at outpatient and inpatient level.

**Fig. 1:**
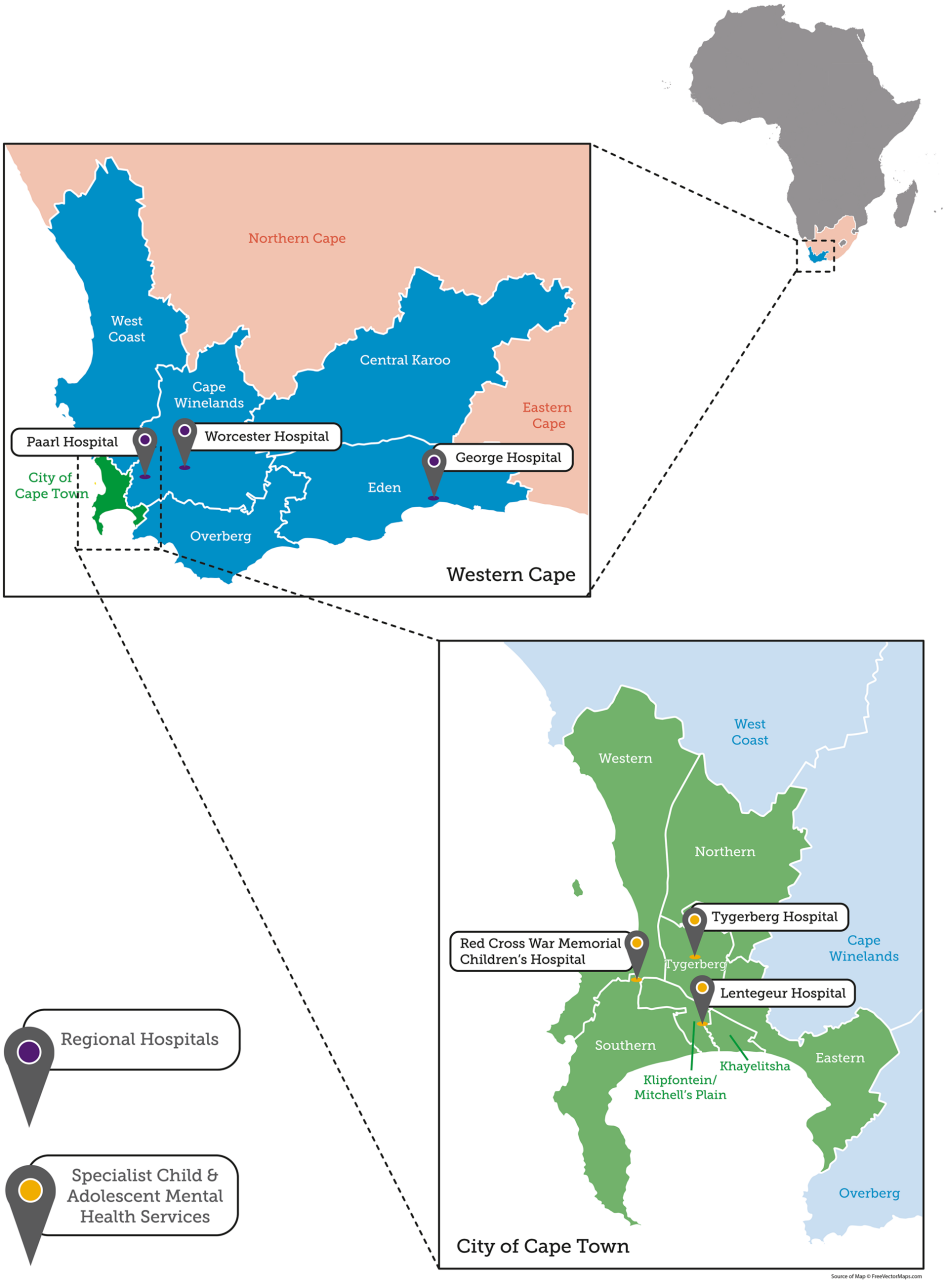
Map of the Western Cape Province showing the metropolitan area (the City of Cape Town and its substructures) and rural health districts. Urban areas are indicated in green; rural areas are indicated in blue. The figure also shows the location of regional hospitals and specialist CAMH units

The Western Cape, one of the nine provinces of South Africa, was the fourth largest both in land size and population at the time of our study. The estimated population was 6.3 million of which 2.1 million (33.9%) were children and adolescents under the age of 19 years. The province has rural and urban areas and is served by one metropolitan municipality (City of Cape Town) and five district councils (West Coast, Cape Winelands, Overberg, Eden and Central Karoo). The metropolitan municipality is divided into four (4) main substructures: The Southern/Western substructure, the Klipfontein/Mitchell’s Plain substructure, the Northern/Tygerberg substructure, and the Khayelitsha/Eastern substructure. Figure 1 shows the province and its urban and rural structures, substructures and districts. In addition, the figure shows the location of specialist CAMH services, and of the regional hospitals in the province.

In 2016, 63.8% of the population lived in the City of Cape Town, followed by the Cape Winelands (13.8%), Eden (9.7%), West Coast (6.9%), Overberg (4.6%), and the Central Karoo (1.2%) [11]. Table 1 shows a more detailed population distribution in the Western Cape by district in 2016. Unfortunately, no disaggregated data were published in the 2016 Census that would allow separation of adolescents (under the age of 19) and younger adults in the 15**–**34-year age groups.

**Table 1:** Population distribution by Western Cape district in 2016 [11]

2016 Community Survey						
	General population		Children, adolescents and young adults			
			0–14 years		15–34 years*	
District/municipality	N	%	N	%	N	%
West Coast	436,403	6.9	113,113	25.9	153,472	35.2
Cape Winelands	866,001	13.8	230,708	26.6	316,210	36.5
Overberg	286,786	4.6	74,764	26.1	94,453	32.9
Eden	611,278	9.7	155,008	25.4	207,010	33.9
Central Karoo	74,247	1.2	18,862	25.4	27,936	37.6
City of Cape Town	4,005,016	63.8	1,042,259	26.0	1,331,960	33.3
Total	6,279,730	100.0	1,521,601	24.23	1,977,569	39.49

*The Census data did not provide any disaggregation between adolescents and younger adults in the 15–34-year age groups

The overall prevalence of CAMH disorders, adjusted for local population factors, comorbidity and according to the risk factors in the Western Cape, were estimated to be 17% [12]. It should be noted that these estimates did not include infant mental health disorders, autism spectrum disorders or neurodevelopmental disorders other than intellectual disability, suggesting that these rates may be an underestimate of the true prevalence of CAMH disorders in the province. Nevertheless, the estimates certainly highlight the burden of CAMH problems in the province, and the need for service provision.

Previous reviews of services and resources suggested a clear neglect of provision for CAMH in the Western Cape [2, 7, 9, 13]. However, as acknowledged by previous authors, due to inadequate information systems, little or no data were available on children and adolescents with mental health problems in primary healthcare settings, general hospital outpatient and inpatient settings, or in specialist CAMH facilities [7, 13, 15].

### Situational analysis and the World Health Organization Assessment Instrument for Mental Health Systems

The World Health Organization (WHO) Assessment Instrument of Mental Health Systems (WHO-AIMS) version 2.2 of 2005 [14] is the WHO framework for conducting situational analyses in LMIC. It was conceptualised and developed by the Mental Health Evidence and Research Team (MER) of the Department of Mental Health and Substance Abuse (MSD) at the WHO, in collaboration with colleagues inside and outside the WHO. The tool is used to collect information on mental health systems with the goal of providing baseline data that can be used to improve mental health systems and to monitor change. The WHO-AIMS framework includes six domains, 28 facets and 156 items to cover key aspects of mental health systems. It includes a brief version that can be used to collect data on a reduced number of variables about mental health systems. Both versions of the WHO-AIMS were developed with general mental health services (as opposed to CAMH) in mind. In fact, the WHO-AIMS framework has only one item specifically on CAMHS (Domain 2, item 2.2.6/Brief version item B13) on the number of children 18 years or younger treated at mental health outpatient facilities, including the proportion of under-19-year-olds treated in comparison to all patients treated. In South Africa, the WHO-AIMS Brief version was previously used to assess CAMH at national level [2] and has been used to assess general mental health services at a regional/provincial level [15].

Here, we therefore set out to perform a fine-grained situational analysis of CAMH in the Western Cape province. Whilst we firmly acknowledge that child and adolescent mental health problems cut across all systems and sectors, for the purpose of this study, given the time and resources constraints, this situational analysis limited its scope to focus on the health system and mainly the Government Department of Health (DoH) public health system, rather than on other or multiple sectors.

## Methods

### Study design

This was a descriptive situational analysis of CAMH services and systems in the Western Cape Province of South Africa.

### Data collection

The Brief version of items from the WHO-AIMS version 2.2 of 2005 was adapted to collect data on CAMHS. We selected 37 items of direct relevance and modified them to focus on CAMH. Table 2 shows the WHO-AIMS items adapted for this situational analysis. Data were collected for the calendar year January to December 2016. At the time of the study, 2016 was the most recent calendar year for which complete public health systems data were available. The situational analysis focused on the DoH and the public health sector, and not on the private health sector or other government departments. WHO-AIMS checklists/survey questionnaires were distributed to key stakeholders in the provincial DoH. Based on the WHO-AIMS table of data sources, relevant documents and records were requested from regional offices, and from individual hospital/clinical facilities in order to complete the questionnaires and fill in any data gaps. The research team established and maintained contact with key stakeholder groups to follow up on the questionnaires, to identify and assist with any difficulties pertaining to the information required, and to verify the information provided. In addition, electronic searches for relevant information through provincial websites and Web of Science (version 5.34) were performed. After collation of all data, further clarifications were sought between January and May 2020 as a final validation check for any potential additional data that may have become available before preparation of this manuscript.

**Table 2:** Variables of interest adapted from the WHO-AIMS version 2.2 (Brief version) for this situational analysis [14]

WHO-AIMS Domains	Western Cape provincial data collected
1. Policy and legislative framework	1.1 CAMH policies, plans, and legislations (B1, B3, B4) 1.2 Human rights legislation relevant to children and adolescents (B5) 1.3 Financing: Expenditure on CAMH by the provincial DoH (B6)
2. Clinical services for children and adolescents with mental health disorders	2.1 Existence and functions of a regional CAMH authority (B9) 2.2 Organisation of CAMH services in terms of catchment areas (B10) 2.3 Outpatient services: Availability of CAMH outpatient facilities, and number/proportion of children and adolescents treated for mental health problems through outpatient facilities at primary, secondary and tertiary levels of care (B11, B12, B13) 2.4 Inpatient services: Availability of CAMH inpatient facilities, and number/proportion of children and adolescents treated (B15, B16, B17) 2.5 Availability of CAMH day patient facilities, community residential facilities, forensic facilities, or CAMH hospitals (B14, B18, B19, B25) 2.6 Interventions (medications): Psychotropic medicines appropriate for children and adolescents included on the essential medicines list; free access to essential psychotropic medicines, and availability of medicines in outpatient and inpatient settings at secondary and tertiary levels of care (B2, B8, B28, B29) 2.7 Interventions (psychosocial): Access to psychosocial interventions in outpatient and inpatient settings at secondary and tertiary levels of care (B26, B27)
3. CAMH in primary healthcare	3.1 Refresher training in CAMH provided to PHC doctors, nurses or other staff and interaction of PHC with specialist CAMHS (B31–B35) 3.2 Availability of medicines and psychosocial interventions in PHC facilities (B27, B33)
4. Human resources	4.1 Human resources in CAMHS (B38–B41)
5. Public education and links with other sectors	5.1 Public education and awareness campaigns about CAMH (B47)
6. Monitoring and research	6.1 Monitoring CAMH (B52, B53) 6.2 Research in CAMH (B54)

CAMH = child and adolescent mental health; B items listed in brackets e.g. (B1) refer to items as listed in the WHO-AIMS Brief Version

### Data capturing and analysis

All data sources were collated and numbered as primary sources of findings. Data sources and data source numbers (DSN) are shown in Table 3. Data were captured on the WHO-AIMS Excel Data Entry program version 2.2 of 2005 for analysis. Analysis was performed through descriptive statistics of variables of interest as outlined in Table 2.

**Table 3:** Data sources for the situational analysis

Data Source Number (DSN)	Data source	Website link	References
DSN01	Policy guidelines. Child and Adolescent Mental Health, 2003	http://www.health.gov.za/index.php/shortcodes/2015-03-29-10-42-47/2015-04-30-08-29-27/mental-health?download=615:policy-guidelines-on-child-and-adolescent-mental-health	[16]
DSN02	Healthcare 2030. The Road to Wellness, Western Cape Department of Health	https://www.westerncape.gov.za/assets/departments/health/healthcare2030.pdf	[17]
DSN03	Mental Health Act no.17 of 2002. National Department of Justice	http://www.justice.gov.za/legislation/acts/2002-017_mentalhealthcare.pdf	[18]
DSN04	Child Care Act 74 of 1983, National Department of Social Development	https://www.westerncape.gov.za/assets/departments/social-development/child_care_act_74_of_1983.pdf	[19]
DSN05	Western Cape Provincial Deputy Director for Mental Health and Substance Abuse	Interview on 9 February 2017 (data available from the author)	[20]
DSN06	Provincial Mental Health Directory, Department of Health, 2015	https://pmhp.za.org/wp-content/uploads/DoH-Mental-Health-Resource-Directory-2015.pdf	[21]
DSN07	Budget 2016 Summary, Western Cape Department of Health	https://www.westerncape.gov.za/assets/departments/treasury/Documents/Budget/2016/2016_budget_summary_budget_day_3_march_2016.pdf	[22]
DSN08	Budget Overview of Provincial Revenue and Expenditure 2016, Treasury of the Western Cape Government	https://www.westerncape.gov.za/assets/departments/treasury/Documents/Budget/2016/2016_overview_of_prov_rev_exp_march_web.pdf	[23]
DSN09	Budget Estimates of Provincial Revenue and Expenditure 2016, Treasury of the Western Cape Government	https://www.westerncape.gov.za/assets/departments/treasury/Documents/Budget/2016/2016_estimates_prov_rev_exp_march_2016_incl_addendums.pdf	[24]
DSN10	Mental Health Services in the Western Cape, Western Cape Department of Health	https://www.westerncape.gov.za/general-publication/mental-health-services-western-cape	[25]
DSN11	Division of Child and Adolescent Psychiatry (DCAP), Western Cape Department of Health	https://www.westerncape.gov.za/general-publication/division-child-and-adolescent-psychiatry-dcap	[26]
DSN12	Mental Health Hospital Services, Western Cape Department of Health	https://www.westerncape.gov.za/service/mental-health-hospital-services	[27]
DSN13	Catchment Areas for Tertiary Child and Adolescent Psychiatry Units in the Western Cape	Data provided by the Head of Clinical Unit, Division of Child and Adolescent Psychiatry (12 March 2020) (data available from the author)	[28]
DSN14	Western Cape Mental Health Data and Facilities List 2016, Western Cape Department of Health	Mental Health provincial information system. The information is not publicly available but was provided by the Provincial Data Management Office for the purposes of this study (data available from the author)	[29]
DSN15	Tygerberg Hospital Annual Report 2016, Western Cape Department of Health	https://www.westerncape.gov.za/sites/www.westerncape.gov.za/files/tygerberg_hospital_annual_report_2016_web.pdf	[30]
DSN16	Court Diversion in the Western Cape Province	https://www.westerncape.gov.za/general-publication/what-diversion	[31]
DSN17	Standard treatment guidelines and essential medicines list for South Africa. Hospital level paediatrics, 2017 edition	http://www.health.gov.za/index.php/standard-treatment-guidelines-and-essential-medicines-list/category/456-hospital-level-paediatrics	[32]
DSN18	First 1,000 Days Campaign, Western Cape Government	https://www.westerncape.gov.za/general-publication/first-1-000-days-campaign	[34]
DSN19	2016 Annual Report, Salesian Life Choices	https://www.lifechoices.co.za/sites/default/files/2017-10/lc_annual_report_2016-final_update_2.pdf	[35]
DSN20	How to handle bullying, Western Cape Education Department	https://www.westerncape.gov.za/general-publication/how-handle-bullying	[36]
DSN21	16 Days of Activism for no violence against women and children, Western Cape Department of Social Development	https://www.westerncape.gov.za/general-publication/what-16-days-activism	[37]
DSN22	Web of Science (version 5.34) data search, April 2020	(articles added in the reference list)	[38–64]

## Results

Below we outline the results for each of the variables of interest shown in Table 2. Each finding includes one or more references as links to the evidence for the finding.

### WHO-AIMS Domain 1: policy and legislative framework

#### Policies, plans and legislations

There was no provincial CAMH policy document and no provincial plan to give effect to the national CAMH policy [16, 17]. There was a general healthcare policy [17] which did not focus on CAMH. Interestingly, the policy acknowledged the need to separate CAMH from adult psychiatric services, but did not present any specific plan to do so. There was no province-specific CAMH mental health legislation. The National Mental Health Act no. 17 of 2002 [18] and the Child Care Act 74 of 1983 [19] were used as legal frameworks in the province [20].

#### Human rights legislation relevant to children and adolescents

To ensure that the human rights of all people (including children and adolescents) were met, a human rights review body (Mental Health Review Board) existed. The Review Board is nominated in terms of section 20 of the Mental Health Care Act 17 of 2002. It consists of three to five members (a mental healthcare practitioner, a magistrate, a South African lawyer and a member of the community). Members should be South African citizens and are appointed by the relevant member of the Executive Council in each province. The Review Board has powers and functions to conduct annual periodic inspections and reviews commissioned by the provincial Minister of Health [18]. Its functions included acting as licensing directorate for non-profit organisations, auditing of mental health programmes, and overall monitoring of mental health practices in the country [20, 21]. All specialist CAMH units had at least one annual external review/inspection of human rights protection of patients. In addition, all specialist CAMH inpatient units had admission, discharge, and seclusion policies, complaints and appeals processes, and procedures to ensure the protection of human rights [20].

#### Expenditure on child and adolescent mental health services by the Provincial Department of Health

There was no separate budget for CAMH, and no budgetary information was presented in a way that could allow disaggregation of adult versus CAMH budgets [22–24]. Primary healthcare budgets were integrated into the overall District Health Services (Community Health Centres and Community-based services) budget. Secondary care budgets were integrated into District Hospital budgets. Tertiary care budgets were integrated either into Provincial Hospital Services (specialised psychiatric hospitals where some tertiary CAMH specialist units were situated) or into Central Hospital Services (central and tertiary hospitals where two tertiary CAMH specialist services were situated). CAMH services were delivered to outpatients at primary care, to outpatients and emergency inpatients at secondary level of care, and to outpatients, emergency inpatients and longer-term inpatients at tertiary level of care.

As shown in Table 4 the overall health services budget in the province for 2016/2017 was just under R20 billion (just over US$1 billion). Of this amount, ~ R7.8 billion (US$ 511.1 million) (39.2%) was allocated to District Health Services, ~ R5.6 billion (US$371.8 million) (28.5%) to Central Hospital Services, ~ R3.2 billion (US$ 208.9 million) (16%) to Provincial Hospital Services, of which ~ R589 million (US$38.5 million) (2.9% of the total health budget) was allocated to mental hospitals [22–24]. Given that none of these groupings provided services exclusively to children and adolescents with mental health problems, it was not possible to identify any CAMH-specific expenditure.

**Table 4:** Western Cape provincial budget for 2016/17 [22–24]

Total health budget in South African Rand	Primary and secondary level	Tertiary level		
	District Health Services	Provincial Hospital Services		Central Hospital Services
R19.983 billion (US$1.307 billion)	R7.826 billion (US $511.140 million) (39.2%)	R3.199 billion (US$ 208.849 million) (16%)		R5.697 billion (US$371.82 million) (28.5%)
		*Non-mental health services*	*Mental health hospitals*	
		R2.610 billion (US $170.427 million) (13.1%)	R589 million (US$38.505million) (2.9%)	

R = South African Rand; At the time of submission (Aug 2020), R1 was equivalent to US$0.058

### WHO-AIMS Domain 2: child and adolescent mental health resources

#### Existence and functions of a regional child and adolescent mental health authority

There was no provincial authority exclusively for CAMH and no provincial director for mental health [20]. There was a provincial deputy-director for mental health and substance abuse. The role of the post-holder was to coordinate all mental health services (including CAMH) within the province to (a) ensure development and implementation of the national policy and legislation, (b) oversee monitoring of services, (c) facilitate equitable budgets for mental health, (d) work closely with district health managers, all stakeholders and sectors, and (e) evaluate services and policy implementation. The deputy-director reported to the provincial director of health programmes [20].

#### Organisation of child and adolescent mental health services in terms of catchment areas

CAMH  services were provided based on where families lived in the province, referred to as geographical service areas (GSAs) or ‘catchment’ areas, as shown in Fig. 1. Services were provided across three levels of care: primary (level 1), secondary (level 2) and tertiary (level 3). The basic patient flow for service organisation was for children and families to start their CAMH ‘journey’ at level 1 services (their catchment area primary healthcare clinic), where they would be seen by a mental health nurse and, if required, a medical officer (a generally-trained doctor). When primary healthcare teams did not feel able to diagnose or treat the child and family, they would be referred to level 2 (their catchment area district hospital) where they would be seen by a general mental health nurse and, if required, a general psychiatrist. At level 2 children and families may also be seen by paediatric services depending on the reason and pathway for referral. When level 2 teams did not feel able to diagnose or treat the child and family, they would refer them to level 3 services. These are services with multidisciplinary expertise in child and adolescent mental health, including child mental health nurses, psychologists with expertise in child mental health, and subspecialist child & adolescent psychiatrists. We refer to level 3 services as ‘specialist CAMH’. Each specialist CAMH team served a specific metropolitan catchment area, specific district hospitals in that catchment area, as well as a specific rural area and its regional hospital. Table 5 shows the catchment areas for each specialist CAMH unit [25–27].

**Table 5:** Catchment areas for specialist child and adolescent mental health services [28]

Specialist CAMH unit	Metropolitan (urban)		Rural	
	Metro catchment area	District hospitals	Rural catchment area	Regional hospital
Division of Child and Adolescent Psychiatry (DCAP)	Southern Western Portion of Klipfontein	New Somerset Hospital Victoria Hospital False Bay Hospital Groote Schuur Hospital Red Cross District Service	Eden	George Hospital
Tygerberg Child and Adolescent Psychiatry team	Northern Tygerberg Portion of Eastern	Karl Bremer Hospital Eerste River Hospital Tygerberg Hospital district service	Portion of Cape Winelands West Coast	Paarl Hospital
Lentegeur Child and Family Unit (CFU)	Khayelitsha Mitchell’s Plain Portion of Eastern Portion of Klipfontein	Khayelitsha Hospital Mitchell’s Plain Hospital Helderberg Hospital	Overberg Central Karoo Portion of Cape Winelands	Worcester Hospital

In 2016 there were three specialist CAMH service units, all based in the metropolitan municipality. The Division of Child and Adolescent Psychiatry (DCAP) (University of Cape Town) was based at Red Cross War Memorial Children’s Hospital in the Southern substructure, the Tygerberg Child and Adolescent Psychiatry team (Stellenbosch University) was based at Tygerberg Hospital, a general tertiary hospital in the Northern substructure, and the Lentegeur Child and Family Unit (University of Cape Town and Stellenbosch University) was based at Lentegeur Hospital, a mental hospital in the Mitchell’s Plain substructure [25–27].

The specialist CAMH service units offered outpatient services to children and families in their catchment areas. Children and adolescents with any mental health problem could be referred by a specialist (psychiatrist or paediatrician) from a relevant catchment area [21, 25–27].

In the time period of this study (January–December 2016), the three units had areas of particular expertise: autism spectrum disorder and infant mental health at DCAP, neuropsychiatric conditions at Tygerberg, and substance abuse and rehabilitation at Lentegeur. Specialist inpatient services were available at each of the three specialist CAMH units but were not based on catchment areas. Instead, they were based on age and/or clinical profile of the child or adolescent. For instance, DCAP had an inpatient unit for children under 12, while Tygerberg and Lentegeur Hospitals had adolescent inpatient units [25–27].

There were also two units that could admit adolescents with intellectual disabilities, one based at Alexandra Hospital, a mental hospital for people with intellectual disabilities, and the other at Lentegeur Hospital. Both these hospitals were in the metropolitan municipality. The units offered inpatient and outpatient services for older adolescents with dual diagnosis (intellectual disabilities and a mental health problem) alongside adults with intellectual disabilities, and served all the districts of the Western Cape. The exclusion criteria for these units were social problems in the absence of intellectual disabilities and comorbid mental health problems, and patients with forensic histories [21, 25–27].

Even though catchment areas and GSAs were clear for general outpatient referrals at primary and secondary levels of care, this was not the case for particular areas of expertise at tertiary level of care, or for inpatient referrals at tertiary level. In addition, there were no specialist CAMH services in any of the rural districts [25–30].

#### Availability of child and adolescent mental health outpatient facilities, and number/proportion of children and adolescents treated for mental health problems through outpatient facilities at primary, secondary and tertiary levels of care

Outpatient services for children and adolescents with mental health problems were available at 317 health facilities spanning primary to tertiary levels of care. Services included limited mobile services and satellite clinics. Only three of all outpatient services (0.9%) were providing dedicated mental health services to children and adolescents. All other facilities (99.1%) were open to all ages [29].

*Primary care (level 1):* There were 273 primary healthcare facilities (206 clinics, 58 community daycentres and 9 community health centres) that provided general mental health services, where children and adolescents were seen alongside adults [29]. Out of a total of 188,369 patients seen for mental health problems at primary level of care, 8300 (4.4%) were children and adolescents. Table 6 shows the number of children and adolescents treated in 2016 in primary healthcare facilities per geographic service area. The majority of children and adolescents were treated in the City of Cape Town (6300/8300; 75.9%) followed by the Eden District and the Cape Winelands. The Central Karoo had the lowest number and proportion of children and adolescents seen for mental health disorders [29].

**Table 6:** The number and proportion of children and adolescents seen in 2016 in primary healthcare (level 1) outpatient settings for mental health problems in the Western Cape [29]

Geographic service areas	Age distribution		Total (% children)
	> 18 years	< 18 years	
City of Cape Town	131,836	6330	138,166 (4.58%)
Cape Winelands District	16,986	609	17,595 (3.46%)
Central Karoo District	3066	49	3115 (1.57%)
Eden District	16,347	850	17,197 (4.94%)
West Coast District	11,834	462	12,296 (3.76%)
Total	180,069	8300	188,369 (4.4%)

*Secondary care (level 2):* Mental health services were provided in 34 district hospitals in the province in 2016 [29]. There were no separate outpatient CAMH facilities at secondary level of care [21, 29] children and adolescents were therefore seen alongside adult psychiatric patients. Out of 15,755 patients seen for mental health problems at secondary level in 2016, 1145 (7.27%) were children or adolescents. Table 7 shows the numbers and proportion of children and adolescents seen at outpatient departments per GSAs in 2016 [29]. The largest number of children and adolescents were seen in the City of Cape Town (561 of 1145; 48.9%), followed by the Eden District and the Cape Winelands. However, as a proportion of all mental health cases, all of the rural districts saw a greater proportion of under 19’s, all in excess of 10% of patients seen (see Table 7).

**Table 7:** The number and proportion of children and adolescents seen in 2016 in secondary care (level 2) outpatient settings for mental health problems in the Western Cape [29]

Geographic service area	Age distribution		
	> 18 years	< 18 years	Total (% children)
City of Cape Town	10,242	561	10,803 (5.19%)
Cape Winelands District	1083	132	1215 (12.19%)
Central Karoo District	162	29	191 (15.18%)
Eden District	2064	248	2312 (10.73%)
West Coast District	1059	175	1234 (14.18%)
Total	14,610	1145	15,755 (7.27%)

*Tertiary care (level 3):* Three specialist CAMH services in the province provided dedicated multidisciplinary outpatient services in 2016. Adolescents with intellectual disabilities and co-occurring psychiatric disorders were seen in the two specialist units for people with intellectual disabilities [25–27].

**Fig. 2:**
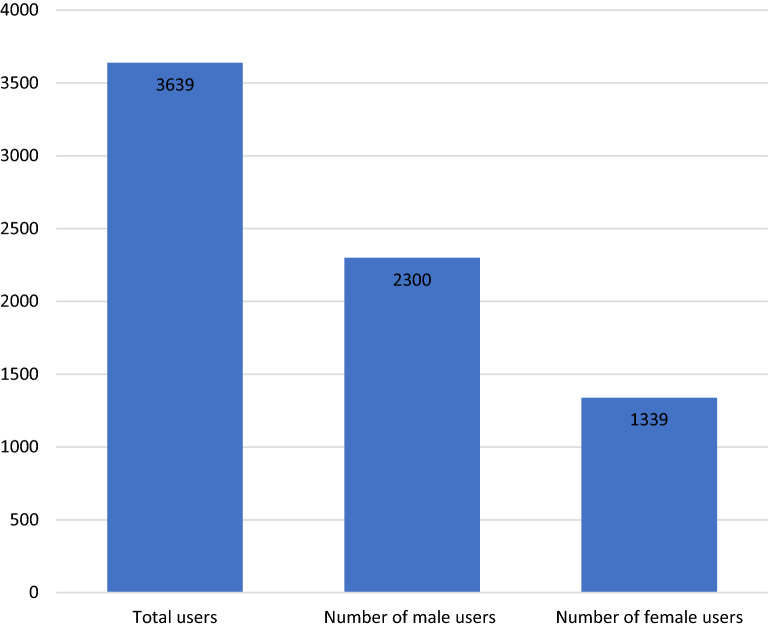
Number and sex of children and adolescents treated as outpatients in the Division of Child and Adolescent Psychiatry, University of Cape Town in 2016

**Fig. 3:**
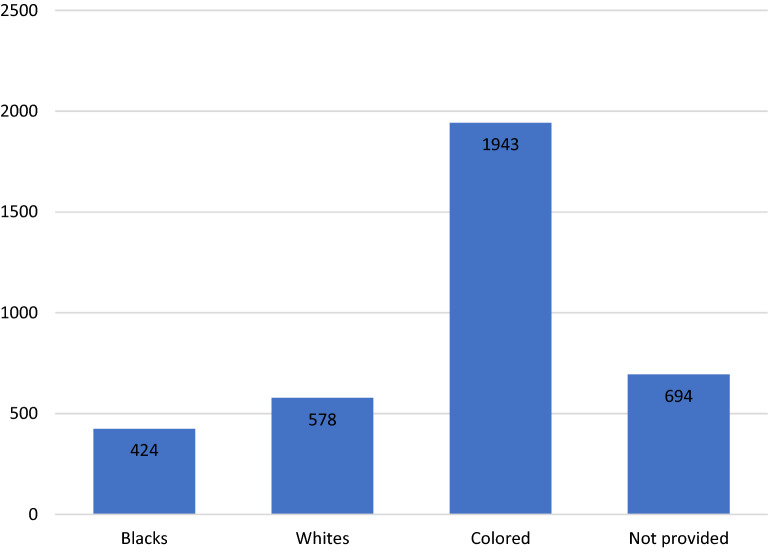
Self-declared ethnicity of children and adolescents treated as outpatients in the Division of Child and Adolescent Psychiatry, University of Cape Town in 2016

No formal summary data were available for Tygerberg and Lentegeur outpatient units in 2016. Patient numbers at DCAP are shown in Fig. 2, and self-declared ethnic/racial distribution in Fig. 3 [28]. A total of 3639 children and adolescents were seen at DCAP in 2016. The majority of patients were male (63.2%), and the self-declared ethnicity (where provided) was coloured (1943/2945; 65.98%), followed by white (578/2945; 19.63%) and black (424/2945; 14.29%).

The Alexandra and Lentegeur units for intellectual disabilities respectively treated three and 39 children and adolescents with intellectual disabilities and co-occuring mental health disorders as outpatients in 2016 [29].

#### Availability of child and adolescent mental health inpatient facilities, and number/proportion of children and adolescents treated

Child and adolescent inpatient services in 2016 were provided at secondary (level 2) and tertiary (level 3) levels. Emergency cases requiring 72-h observations were admitted at secondary level and medium to long-term admissions were provided at tertiary level [29].

*Secondary care (level 2):* Secondary level services were available in 42 health facilities [29]. There were no dedicated beds for children and adolescents with mental health problems in these hospitals. Children and adolescents who required inpatient emergency admission were admitted to paediatric units (up to age 12) or adult psychiatric wards (13–18 years). There were no disaggregated data for children and adolescents who were admitted to paediatric units or adult psychiatric wards [29]. Table 8 shows the numbers of children and adolescents per geographic service area who were admitted in level 2 inpatient emergency units in 2016 [29]. A total of 795 children and adolescents, representing 4.35% of all secondary care mental health admissions, were admitted. The majority of children and adolescents were treated in the City of Cape Town (473/795; 59.5%), followed by the Cape Winelands and Eden Districts (see Table 8). Interestingly, the rural districts had more than double the proportion of under-18-year-old admissions relative to total population numbers, compared to the City of Cape Town. In the City of Cape Town 3.31% of level 2 admissions were under the age of 18, while in rural districts the proportions were 7.27–9.66% [29].

**Table 8:** Children and adolescents with mental health problems admitted to secondary care (level 2) inpatient facilities per geographic service area in the Western Cape [29]

Geographic service area	Age distribution		Total (% children)
	> 18 years	< 18 years	
City of Cape Town	13,824	473	14,297 (3.31%)
Cape Winelands District	1339	105	1444 (7.27%)
Central Karoo District	131	14	145 (9.66%)
Eden District	889	82	971 (8.44%)
Overberg District	487	42	529 (7.93%)
West Coast District	795	79	874 (9.03%)
Total	17,465	795	18,260 (4.35%)

*Tertiary care (level 3):* Children and adolescents were admitted to one of three specialist CAMH inpatient units or to one of two intellectual disabilities inpatient units. Where this was not possible, admission to adult mental health units were made. All tertiary inpatient beds were in the City of Cape Town. A total of 346 children and adolescents were admitted to specialist inpatient units in 2016 [29]. The breakdown of admissions is outlined below.

The inpatient unit at DCAP (known as the Therapeutic Learning Centre) was a 6-bedded unit for 6–12-year-olds with complex or severe mental health problems, where children are typically admitted for 3–6 months. The unit admitted a total of 26 patients in 2016. The Tygerberg Child and Adolescent Psychiatry unit had a 16-bedded adolescent inpatient unit. Adolescents were admitted either for short-term diagnostic assessment or for longer-term intervention. The unit admitted 157 patients in 2016. The Lentegeur Child and Family Unit had an 8-bedded adolescent unit, and also admitted adolescents for short-term diagnostic work-up, or short to medium-term therapeutic work. The unit admitted 152 patients in 2016 [29]. Eleven children or adolescents were admitted to adult mental health hospitals: Valkenberg = 8; Alexandra = 2; Stikland = 1 [29].

#### Availability of child and adolescent mental health day facilities, community residential facilities, forensic facilities or hospitals

##### Community or other residential facilities

There were no Department of Health day facilities and residential facilities exclusively for children and adolescents with mental health problems [29]. The Department of Social Development provided community residential facilities for children and adolescents with a range of psychosocial challenges including abuse, neglect or other social difficulties. Children and adolescents with mild mental health problems who needed care, were placed in these units. There were 69 of these facilities listed in the Western Cape in 2016 [29], but no data were available on the number of children in these facilities during the year. There were also special care centres and licensed homes for children and adults with severe and profound intellectual and physical disabilities, but no child and adolescent data were available [21].

##### Child and adolescent forensic and other residential facilities

Child and adolescent forensic mental health services were provided at one of the adult mental hospitals (Valkenberg Hospital) in the City of Cape Town [28]. A consultant psychiatrist, two child & adolescent psychiatrists, and five clinical psychologists provided sessions to the service. CAMH forensic services were aimed at providing the Department of Justice with criminal capacity/forensic psychiatric assessments for children in conflict with the law [30]. Court diversion programmes were also available to provide support for 10–18-year-olds who were both in conflict with the law and had co-occurring mental health needs. These therapeutic programmes were facilitated by a probation officer or social worker and were offered for a period of 3–12 months. Programmes were provided by the Department of Social Development and a range of non-governmental/non-profit organisations [31].

##### Child and adolescent mental hospitals

There were no dedicated CAMH hospitals in the province in 2016 [21, 25].

#### Interventions (medications): psychotropic medicines appropriate for children and adolescents included on the essential medicines list, free access to essential psychotropic medicines, and availability of medicines in outpatient and inpatient settings at secondary and tertiary levels of care

All mental health facilities (outpatient and inpatient) at secondary and tertiary levels of care had at least one psychotropic medicine of each therapeutic class (antipsychotic, antidepressant, mood stabiliser, anxiolytic, stimulant and anti-epileptic medicines) available [28]. These drugs were fluoxetine, citalopram, haloperidol, chlorpromazine, imipramine, risperidone, methylphenidate, lorazepam, lithium carbonate, sodium valproate, carbamazepine, citalopram and biperiden [32]. About 4.8 million people including children and adolescents (76%) in the Western Cape qualified for free access to healthcare services and for those with mental health disorders, to essential psychotropic medicines, if required [24]. As outlined earlier, healthcare is provided free of charge to people without private health insurance, to those with household income below ZAR100,000 per year, to all children under the age of six, and to people with disabilities. A sliding scale is used to calculate the costs of the service [33].

#### Interventions (psychosocial): access to psychosocial interventions in outpatient and inpatient settings at secondary and tertiary levels of care

Secondary level outpatient and inpatient services provided very limited access to psychosocial interventions for children and adolescents [28]. All specialist CAMH units provided multidisciplinary care at outpatient and inpatient level. This involved access to a range of psychosocial interventions appropriate for children and adolescents including psychoeducation, family interventions, counselling, individual psychological therapies, play-based and other evidence-based psychosocial interventions [16, 29].

### WHO-AIMS Domain 3: child and adolescent mental health in primary healthcare

#### Refresher training in child and adolescent mental healthcare provided to primary healthcare doctors, nurses or other staff and interactions of primary healthcare with specialist child and adolescent mental health services

The WHO-AIMS specifically seeks to quantify the proportion of mental health training to primary care doctors, nurses and other primary health care (PHC) staff in relation to other training provided in the year. The document also specifically seeks to quantify the number of primary healthcare staff that have received at least 2 days of refresher training in mental health in the last year. No data were available to answer these specific questions. However, as actors in CAMH, we know that specialist CAMH teams provided outreach and support to colleagues at primary care level, including to medical doctors and nurses. Training was provided through workshops and seminars, and clinical consultation through a range of modalities including face-to-face, telephonic consultations, informal meetings, review of individual cases or through discussion of referral issues. However, there was no formal documentation of informal interactions with primary care staff in any health information systems, and the majority of these opportunities were with primary care colleagues in the metropolitan area [26, 30].

#### Availability of medicines and psychosocial interventions in primary healthcare facilities

All primary healthcare facilities had at least one psychotropic medicine of each therapeutic class (antipsychotic, antidepressant, mood stabiliser, anxiolytic, stimulant and anti-epileptic medicines) available [20]. The majority of the population had free access to medications. There was little if any access to psychosocial interventions for children or adolescents available at primary care level [28].

### WHO-AIMS Domain 4: human resources

#### Human resources in child and adolescent mental health services

**Fig. 4:**
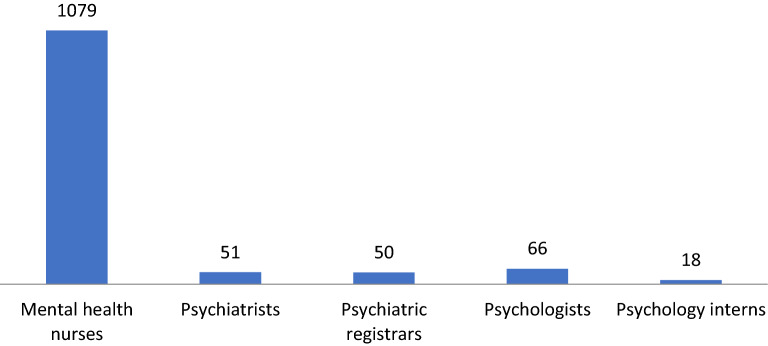
Profile of mental health services staff in the Western Cape in 2016

No formal data were available on human resources specifically for CAMH. The majority of mental health human resources data represented staffing for all specialties across all ages. There were also no differentiation of data between staffing for outpatient versus inpatient care. Figure 4 shows the overall mental health human resources in the Western Cape in 2016. The majority of staff were mental health nurses (n = 1079; 85.4% of all mental health staff), and there were few specialist psychiatrists (n = 51; 4.03% of all mental health staff). Eight of the 51 psychiatrists (15.7%) were qualified as child & adolescent psychiatrists. Trainees (psychiatric registrars and psychology interns) represented 5.38% of the workforce) [29].

**Fig. 5:**
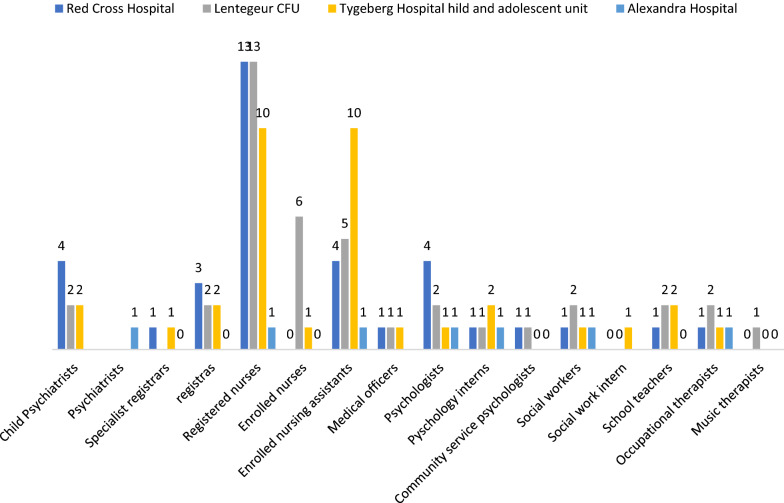
Profile of mental health services staff in specialist child and adolescent mental health units in the Western Cape in 2016

In the absence of provincial human resources data for specialist CAMH, data were collected directly from the three tertiary CAMH units [29]. Figure 5 provides best estimates of human resources in the specialist CAMH facilities. As shown in Fig. 5 all these units had multidisciplinary teams which included child & adolescent psychiatry, psychology, nursing and other disciplines, but there was significant variability in the workforce profile between the specialist units. Psychiatrists and psychologists were appointed on contracts with ‘joint conditions’ between the Western Cape Government and respective universities, and were allocated 70% of their time to deliver clinical care and 30% of their time for academic activities (including teaching, supervision and research). Staff in other categories were appointed by the Western Cape Government with little to no time dedicated for teaching/ training, supervision and research.

In 2016, there were three posts in the province for psychiatrists to train as child & adolescent psychiatrists. However, there were no vacant positions in the government sector for child & adolescent psychiatrists once qualified. There was therefore no ‘pipeline’ that could support new child & adolescent psychiatrists to join the state sector. Newly qualified child & adolescent psychiatrists had to move into private practice, work in general psychiatry posts, or leave the country. There were no funded training programmes for psychologists, nurses, occupational therapists, or speech and language therapists to specialise in child and adolescent mental health.

### WHO-AIMS Domain 5: public education and links with other sectors

#### Public education and awareness campaigns about child and adolescent mental health

There were no formalised intersectoral collaborations between the DoH and other sectors specifically related to child and adolescent mental health and mental illness in children or adolescents [20].

One initiative, led by the Department of Social Development (DSD) and formally adopted by all provincial government departments, was related to infant mental health. The ‘First 1000 Days of Life’ project was a collaborative cross-agency programme involving the DoH, DSD and the Western Cape Education Department (WCED) and was set up to identify and reduce risk factors for maternal and infant mental health problems in the first 1000 days of life [34].

A number of other projects and campaigns were aimed at promoting child and adolescent well-being. These included a competition launched by the Salesian Life Choices (a youth organisation) to encourage young people to start discussions around health issues and depression [35], an anti-bullying campaign in schools run by the WCED in May 2016 [36] and the International 16 Days of Activism, a campaign aimed at increasing awareness of the negative impact of violence and abuse against women and children [37].

### WHO-AIMS Domain 6: monitoring and research

#### Monitoring of child and adolescent mental health services

There was a formally defined list of individual data items that ought to be collected by all child and adolescent mental health facilities, using CLINICOM and SENJANI information systems [20, 29]. CLINICOM is a hospital information system that is used in various countries. It records the activities of a range of clinicians in outpatient and inpatient facilities and the data is saved comprehensively in one place. It keeps records of patients treated in medical facilities, medical documentation, laboratory test results, daily course of diseases, patient medication records, examination scheduling by specialists, rehabilitation and rehabilitation procedures. In South Africa and in the Western Cape, CLINICOM is a computer-based data capture programme used in secondary and tertiary hospitals in the province. All new patients and patient-related activities are expected to be captured on the CLINICOM system by clinic administrators. A sub-component of CLINICOM is used in primary healthcare facilities, to capture data on children and adolescents who present with mental health problems including personal information, inpatient and outpatient attendance, admissions, discharges, clinic, and diagnosis [20]. Despite the apparent availability of these information systems, it was not clear to what extent different healthcare facilities completed or utilised them. It was not possible for us to have direct access to the CLINICOM system to extract CAMH-related data. It was equally surprising that summative data, as presented in annual reports, were never disaggregated to present findings on children and adolescents separately from information on adults. There were no DoH or mental health reports in 2016 that included references to CAMH [20]. To our knowledge, there has never been a dedicated report from the provincial Department of Health on child and adolescent mental health.

#### Research in child and adolescent mental health

The three specialist CAMH units were linked to two universities (University of Cape Town and Stellenbosch University) and provided infrastructure and supervision for research in CAMH. In 2016 there were five research programmes, all led from the University of Cape Town, but with collaborations at Stellenbosch University. The programmes included the Adolescent Health Research Unit, the Centre for Autism Research in Africa, a Tuberous Sclerosis Complex Research Programme, a staff research development programme, and an Infant Mental Health research programme.

We performed a brief literature search for all publications relevant to CAMH published in 2016 by authors from the Western Cape or on participants from the Western Cape. We used Web of Science (version 5.34). Core Collection and the following search terms: “child AND adolescent AND mental AND health AND South Africa”; “autism AND South Africa”; “ADHD AND South Africa”; “adolescence AND South Africa”; “infant AND mental AND health AND South Africa” and “tuberous sclerosis”. We then refined searches by selecting affiliations with institutions in the Western Cape. All identified abstracts were reviewed for relevance to CAMH. A total of 44 abstracts were identified. After removal of duplicates and abstracts not directly relevant to CAMH, a total of 26 articles were included [38–64]. A brief thematic grouping identified two articles on infant mental health, four on autism or ADHD, seven on adolescent mental health and five on tuberous sclerosis complex (TSC). It was of interest that a further six articles related to mental health aspects of HIV/AIDS, with the remaining three focusing on other themes of particular relevance in a South African setting: child abuse, foetal alcohol spectrum disorder (FAS), and cultural adaptation of an instrument to identify psychopathology. Table 9 provides a summary of the research relevant to CAMH and published in 2016 as identified by our search.

**Table 9:** Publications of research relevant to child and adolescent mental health that included authors from the Western Cape or a focus on the Western Cape in 2016

Research theme	Topic of research	Reference (First author, journal, volume: pages)
Infant Mental Health	Infant mental health and early childhood [38]	Worthman, *Social Science and Medicine*, 154: 62–69
	Reflective practice in infant mental health [39]	Berg, *Infant Mental Health Journal*, 37: 684–691
Autism and ADHD	Autism in Africa [40]	de Vries, *Current Opinion in Neurology*, 29: 130–136
	Performance of South African children on the CSBS, a tool for autism [41]	Chambers, *International Journal of Language and Communication Disorders*, 51: 265–275
	Theory of mind in autism [42]	Hamilton, *Journal of Child and Adolescent Mental Health*, 28: 233–241
	Management of ADHD in children and adolescents: clinical audit of ADHD assessment and treatment [43]	Vrba, *Journal of Child and Adolescent Mental Health*, 28: 1–19
HIV/AIDS	HIV-associated neurocognitive disorders in 6–16-year olds [44]	Hoare, *Neurology*, 87: 86–93
	The impact of household HIV on child development [45]	Sherr, *Child Care Health and Development*, 42: 890–899
	Mental health resilience in children who lost parents to HIV/AIDS [46]	Collishaw, *Journal of Abnormal Child Psychology*, 44: 719–730
	Social support for children affected by HIV/AIDS [47]	Sharer, *Aids Care—Psychological and Socio-Medical Aspects of AIDS/HIV*, 28: 110–117
	Resilience in HIV-affected adolescents in South Africa [48]	Bhana, *Aids Care—Psychological and Socio-Medical Aspects of AIDS/HIV*, 28: 49–59
	Correlates of emotional and behavioural problems in children with perinatally-acquired HIV [49]	Louw, *Aids Care—Psychological and Socio-Medical Aspects of AIDS/HIV*, 28: 842–850
Adolescent Mental Health	Mental health inequalities in adolescents [50]	Das-Munshi, *PloS One*, 11: 5
	Adolescent substance abuse [51]	Weybright, *Journal of Adolescence*, 49: 158–169
	Impact of family structure on adolescent psychological profile [52]	Davids, *Journal of Psychology in Africa*, 26: 351–356
	Social protection and adolescent health [53]	Cluver, *PloS One*, 11: 10
	Parenting programme to prevent abuse of adolescents [54]	Cluver, *Trials*, 17: 328
	Reducing adolescent abuse in LMIC [55]	Cluver, *BMC Public Health*, 16: 567
	Factors associated with readmission of adolescents discharged from inpatient units [56]	Pieterse, *Journal of Child and Adolescent Mental Health*, 28: [incomplete]
Tuberous Sclerosis Complex (TSC)	Clinical trial of everolimus for epilepsy in TSC [57]	French, *Lancet*, 388: 2153–2163
	Everolimus for neurocognitive problems in TSC [58]	Randell, *Trials*, 17: 398
	Long-Term use of everolimus for SEGA in TSC [59]	Franz, *PloS One*, 11: 6
	Towards an improved understanding of TSC-Associated Neuropsychiatric Disorders [60]	Leclezio, *Advances in Autism*, 2: 1–8
	Everolimus for renal angiomyolipomas in TSC [61]	Bissler, *Nephrology, dialysis and transplantation*, 31: 111–119
Other themes	Fatal child abuse [62]	Mathews, *South African Medical Journal*, 106: 22–25
	Theory of mind in children with FASD [63]	Lindinger, *Alcoholism—Clinical and Experimental Research*, 40: 367–376
	Cultural adaptation of the DISC-IV for Sotho-speaking South Africans [64]	Skinner, *Journal of Ethic & Cultural Diversity in Social Work*, 25: 1–19

## Discussion

The aim of this study was to perform a fine-grained situational analysis of CAMH services and systems in the Western Cape as a ‘case study’ for similar services in the rest South Africa. In addition, we hoped that the situational analysis may also provide a model for other low/middle-income countries. Items from the WHO-AIMS 2.2 (Brief version) were adapted to capture information about the six fundamental domains of the CAMH health system.

In terms of policy and legislative frameworks for CAMHS (WHO-AIMS Domain 1) there was an absence of any provincial CAMH policies or implementation plans [20]. This finding was in line with an earlier policy analysis conducted by us and others [65, 66]. We identified reasonable human rights policies and procedures for children. Of note was the lack of disaggregated budgets for CAMH. It was therefore not possible to comment on financing of CAMH in the province. The budget for mental hospitals represented only three percent (3%) of the total health budget in the 2016/17 financial year. The CAMH budget would therefore have been a fraction of that three percent (3%).

Policy development reflects commitment from the (provincial) government and relevant authorities towards specific services, and provides a mandate to support funding mechanisms [64]. Lack of this important hardware element for CAMH in the Western Cape therefore raises questions about the commitment of the provincial government and authorities towards strengthening of CAMH. The lack of dedicated funding for CAMH was a second related concern. Our observations are underlined by the findings of Mokitimi and colleagues [67] and Docrat and colleagues [7] who confirmed disproportionately low expenditure on CAMH in comparison to adult mental health services for the same period (January to December 2016). Adequate provision of resources in a health system requires adequate financing to ensure that a good enough service is delivered [4]. Child and adolescent mental health disorders have a ‘triple burden’ in terms of its economic impact—first in terms of the child (costs associated with the treatment of the child, loss of future earnings as a result of disability and reduced work capacity for the individual), second in terms of the caregivers and family (reduced family income as a result of caregiving and loss of productivity), and third in terms of wider societal costs (costs to the healthcare, social care, criminal justice and educational system) [68, 69].

In terms of clinical services for CAMH (Domain 2), there was no dedicated CAMH authority. This signals a lack of dedicated leadership and governance of CAMH. Leadership and governance in a health system is important to ensure coordination of the services and equitable allocation of available resources [4]. Outpatient (but not inpatient) services were broadly organised in terms of catchment areas and across three levels of care (primary, secondary and tertiary). It was with great difficulty that we were able to disaggregate clinical data to identify that 8300 under-18-year-olds were seen for mental health problems at primary, and 1145 at secondary level. This represented 4.4% and 7.27% respectively of all people seen for mental health problems at primary and secondary levels of care. We were only able to find outpatient tertiary data for one of the three specialist CAMH units, where 3639 children and adolescents were seen as outpatients in 2016. A total of 795 under-18-year-olds were admitted to inpatient units in secondary care (representing 4.35% of all secondary care mental health admissions) and a total of 346 in specialist CAMH inpatient units.

It was of concern to observe that all mental health services for children and adolescents at primary and secondary levels of care were mixed with adults. We were particularly concerned by the admission of eleven under-19-year-olds to adult mental hospitals. These findings concurred with the views of senior stakeholders in the system as identified previously by Mokitimi and colleagues [67]. At all levels of care separate CAMHS should exist and adaptations are required to meet the needs of children and adolescents [4, 10]. Adult psychiatric environments are not conducive to the mental health and well-being of children and adolescents. It can jeopardise the safety of young people and can be frightening and traumatising, having a direct negative impact on the quality of care for children and adolescents [66]. International and national guidelines have previously recommended dedicated CAMH services [4, 10] and even the Western Cape General Health Policy [17] set out a plan to separate children and adolescents from adult psychiatric services. However, the findings presented here suggest that no implementation of these guidelines had taken place by the end of 2016.

A total of 63.8% of the Western Cape population lived in the City of Cape Town in 2016 [11]. Of all under-19-year-olds seen in primary care, 76.3% lived in the City of Cape Town. In contrast, at secondary care level, only 48.9% of the same age group outpatients and 59.5% of inpatients were in the metropolitan area. In the rural districts, by contrast, a greater proportion of under-18-year-olds were seen at secondary care facilities. All specialist CAMH units were based in the City of Cape Town. Child & Adolescent Psychiatry is registered as a ‘subspecialty’ in South Africa, and specialist CAMH clinics are therefore based at university teaching hospitals only. All university teaching hospitals in the Western Cape are based in the City of Cape Town, hence the reason that all specialist clinics were based in the city. These findings suggest that the lack of easy and close access to specialist CAMH in the rural districts of the province may have added additional service pressure at the secondary level of care. This would be a very important empirical question to explore in future health systems research.

In terms of access to interventions, levels 1 and 2 primarily provided access to medication treatment, with little or no psychosocial support. Only at specialist CAMH level did children and adolescents have access to a range of medication, psychosocial treatment and the support of multidisciplinary teams.

Domain 3 of the WHO-AIMS focuses on CAMH in primary care (level 1). Even though 8,300 children and adolescents were seen in primary care settings, and despite the fact that there was anecdotal evidence of refresher training on CAMH for primary care staff, there were no sources documenting any access to refresher training on CAMH to PHC staff. As alluded to earlier, primary care staff had access to psychotropic medications on the essential drugs list, but very little if any access to psychosocial support. It is therefore of concern that we were not able to find clear data or evidence of consistent and sustained support to service providers, particularly at primary levels of care, where the majority of children and adolescents will first present with potential mental health problems. This observation is of particular importance in the context of South African prioritisation of public health and primary healthcare services.

It was difficult to access data on human resources for CAMH (Domain 4 of the WHO-AIMS) given that all CAMH staffing data were combined with adult mental health staff data in formal provincial datasets. Direct collection of data from specialist CAMH units allowed the basic description of the multidisciplinary staffing in these units. Data indicated high clinical and teaching activity in specialist CAMH units, but very limited dedicated resources for staff training and capacity-building, for instance, in training posts in CAMH.

In the 2016 data we could only identify one clear example of a joint initiative across Provincial Government Agencies (Health, Education, Social Development, Justice), focused on CAMH. The ‘First 1000 Days of Life’ was cited as a positive initiative around maternal mental and physical health, and infant mental health. There was little evidence of other joint initiatives between sectors (WHO-AIMS, Domain 5). This lack of intersectoral collaboration and inequitable distribution of CAMH resources mirrors findings from a senior stakeholder group [67].

Domain 6 captured information on monitoring and research. Even though all levels of care apparently had information systems to collect data on patient care, we were not able to access any of the primary data collected through the CLINICOM system, and were not able to get access to any data where under-18-year-old mental health activity data were clearly disaggregated from other mental health data. This raised the question about how ‘fit for purpose’ the health information systems in the Western Cape may be for CAMH. Based on our observations, the existing information systems which lacks categories that would allow for disaggregation, may not be able to provide adequate and sufficiently nuanced information to help identify the problem areas in CAMH services, in order to inform the planning for responsive services and programmes [68], or for research on CAMH.

We were encouraged to see active research in CAMH in 2016 across a number of broad thematic domains, and that a significant proportion of CAMH research was on topics of particular resonance in the African context (e.g., HIV/AIDS, FASD, resilience, and child abuse).

Taking together the findings in the situational analysis, we identified a number of the gaps that will require urgent action. Table 10 shows an overview of our findings. Gaps were identified in all health systems domains, and at all levels of the healthcare system. These included the previously identified lack of policy development and implementation for CAMH, lack of resources (financial, governance, infrastructural and human), lack of public education, and lack of access to information services that could support monitoring and research in CAMH.

**Table 10:** Health system gaps in child and adolescent mental health in the Western Cape as identified in this situational analysis

WHO-AIMS Domain	Gaps in CAMHS
*Domain 1 * Policy and legislative framework	• No provincial CAMH policy or implementation plans • No dedicated financing for CAMH
*Domain 2 * Clinical services for children and adolescents with mental health disorders	• No dedicated leadership and governance structure for CAMH • No dedicated CAMH services and lack of psychosocial interventions at secondary level • No specialist CAMH services in rural districts
*Domain 3 * CAMH in primary healthcare	• Inadequate documentation on training of professionals on CAMHS at primary care level • Lack of dedicated resources at secondary and tertiary care levels to support and train colleagues at primary level, particularly in rural districts • Lack of psychosocial interventions at primary care level
*Domain 4* Human resources	• Limited information systems to access human resources data on CAMH • Limited human resources for CAMH at secondary and tertiary care levels
*Domain 5 * Public education and links with other sectors	• Limited public health campaigns on CAMH • Limited intersectoral collaboration about CAMH
*Domain 6* Monitoring and research	• Lack of disaggregated and accessible information systems for CAMH

In recent work by Docrat and colleagues [7], a national survey of mental health costs was performed, using data for the same period as used in our study (2016/17). Apart from finding that the public mental health budget represented only 5% of the overall public health budget, they estimated that ~ 7.5% of the total uninsured population required some form of outpatient mental health care, and ~ 0.89% required inpatient mental health services [7]. Applying the same estimates to CAMH (as a rough calculation), we would have expected 157,500 children in the Western Cape to have required outpatient and 18,690 inpatient care. The data identified in this situational analysis identified a fraction of such expected numbers across all levels of care: 6.3% (9922.5/157,500) of expected outpatient visits and 7.2% (1346/18,690) of expected inpatient admissions. It is therefore very sobering to conclude that existing mental health services across all levels of care reached fewer than 10% of children and adolescents who may have needed mental healthcare in 2016. Of those reached, only those who accessed specialist CAMHS were likely to have received comprehensive evidence-based care.

### Limitations of the study

The main limitation of this situational analysis was challenges of accessing accurate and disaggregated data through existing health information systems. We therefore acknowledge that our findings may not have been representative of the actual CAMH clinical activity and resources in the Western Cape in 2016. However, we tried to be rigorous and systematic in data collection and presented all data sources in a systematic way to optimise the accuracy of data collected. We recognise the lack of clear and accessible information systems as a limitation of our study, but also note this as a major gap in CAMH in the province. We also acknowledge that, given the period of data identified and discussed (January–December 2016), these represent an historical analysis and that some of the findings may have changed since 2016. Finally, we acknowledge that we focused our analysis on the Department of Health to the exclusion of all the other sectors (Education, Social Development, Non-Profit etc.) that are also of fundamental importance in CAMH. Broadening out the focus to other sectors and to inter-sectorality would be a very important area for future CAMH research in the South African context.

### Relevance of findings to other low- and middle-income countries

The WHO-AIMS was developed as a tool for situational analysis of adult mental health services and, in its original form, only had one item about CAMH services. In its original form it was therefore not of particular use to generate a detailed situational analysis of CAMH in any country. We made a range of adaptations to the BRIEF version of the WHO-AIMS in order to collect much more breadth and depth about CAMH resources in our study. To our knowledge no other country has used a similar approach to generate a situational analysis specifically on CAMH. Given the vital importance of CAMH services throughout the world, but particularly in LMIC settings, development of a bespoke framework for CAMH situational analysis may be a very helpful action by the WHO. The version as adapted by us in this study may provide a useful starting place for such a global process.

There is no doubt that child and adolescent mental health (CAMH) services and systems have been neglected over the last number of decades, and countries around the globe are far from seeing parity between physical health and mental health services, despite the global burden of CAMH disorders. In a recent review Simelane and de Vries [68] reviewed health systems mapping and strengthening initiatives in CAMH in low/middle-income countries. They used the WHO-AIMS framework to group these studies. Overall, only a handful of mapping studies were identified over the last few years, mainly in South Africa and India [69]. Across the WHO-AIMS domains some innovations were identified to strengthen specific aspects. Apart from the WHO-AIMS domains, digital technology was identified as a potentially exciting vehicle to strengthen many health system domains. However, the authors argued that any system strengthening should be articulated with a ‘whole-systems’ perspective, mindful of the cultural, socio-economic and linguistic diversity of LMIC communities [69].

## Conclusions

In comparison to most other South African provinces, the Western Cape is seen as relatively well-resourced in terms of health services. In spite of this, our situational analysis identified many hardware systems gaps, including lack of a provincial CAMH policy/implementation plans, lack of dedicated funding, leadership and governance of CAMH, lack of dedicated clinical services at primary and secondary care levels and in rural districts, lack of targeted public health campaigns, limited intersectoral collaboration, and inadequate information systems to provide disaggregated CAMH data. Overall data suggested clinical care provision to fewer than 10% of the expected population of children and adolescents in need of mental healthcare. Our findings raise significant concerns about CAMH in the Western Cape, and by implication, about CAMH services and systems elsewhere in the country and in other low/middle-income countries [2, 10, 13, 15, 65, 66, 69–71]. Even though it will be important to expand our research also to explore software elements of the health system (e.g. through the experience of grassroots level service providers and users), the data presented here are already a clear call for action to find strategies and initiatives that will strengthen CAMH in the province, the country and in other LMIC.

## Data Availability

The majority of data included are publicly available. All other data are available from the authors.

## Abbreviations

CAMH: Child and Adolescent Mental Health
CAMHS: Child and Adolescent Mental Health Services
DCAP: Division of Child and Adolescent Psychiatry
DoH: Department of Health
DSD: Department of Social Development
DSN: Data source numbers
GSAs: Geographical service areas
LMIC: Low- and middle-income countries
PHC: Primary Health Care
WCED: Western Cape Education Department
WHO-AIMS: World Health Organization Assessment Instrument of Mental Health Systems
WHO: World Health Organization
